# A hairy-leaf gene, *BLANKET LEAF*, of wild *Oryza nivara* increases photosynthetic water use efficiency in rice

**DOI:** 10.1186/s12284-017-0158-1

**Published:** 2017-05-12

**Authors:** Norimitsu Hamaoka, Hideshi Yasui, Yoshiyuki Yamagata, Yoko Inoue, Naruto Furuya, Takuya Araki, Osamu Ueno, Atsushi Yoshimura

**Affiliations:** 1 Faculty of Agriculture, Kyushu University, 6-10-1 Hakozaki, Higashi-ku, Fukuoka, 812-8581 Japan; 2 Faculty of Agriculture, Ehime University, Tarumi, Matsuyama, Ehime 790-8566 Japan

**Keywords:** Leaf hair, *Oryza nivara*, Photosynthetic trait, Water use efficiency, Wild rice

## Abstract

**Background:**

High water use efficiency is essential to water-saving cropping. Morphological traits that affect photosynthetic water use efficiency are not well known. We examined whether leaf hairiness improves photosynthetic water use efficiency in rice.

**Results:**

A chromosome segment introgression line (IL-hairy) of wild *Oryza nivara* (Acc. IRGC105715) with the genetic background of *Oryza sativa* cultivar ‘IR24’ had high leaf pubescence (hair). The leaf hairs developed along small vascular bundles. Linkage analysis in BC_5_F_2_ and F_3_ populations showed that the trait was governed by a single gene, designated *BLANKET LEAF* (*BKL*), on chromosome 6. IL-hairy plants had a warmer leaf surface in sunlight, probably due to increased boundary layer resistance. They had a lower transpiration rate under moderate and high light intensities, resulting in higher photosynthetic water use efficiency.

**Conclusion:**

Introgression of *BKL* on chromosome 6 from *O. nivara* improved photosynthetic water use efficiency in the genetic background of IR24.

**Electronic supplementary material:**

The online version of this article (doi:10.1186/s12284-017-0158-1) contains supplementary material, which is available to authorized users.

## Background

Rice is a major staple food around the world. Nearly half of the rice-growing area is rainfed uplands or lowlands (Kato et al. 2008), but the productivity of those fields is generally lower than that of irrigated lowlands. Irrigated lowlands produce 75% of all rice grain, and water-limited areas produce 23% (Maclean et al. 2013). Therefore, effective use of water resources is an important target for sustainable rice cropping.

In adapting to new environments, plants gain or lose ecological, morphological, physiological, and anatomical traits (Lande 2009; Nicotra et al. 2010). Many higher plant species have leaf hairs, called glandular or non-glandular trichomes (Zeng et al. 2013). These leaf hairs have important roles such as protection from herbivory by insects and infection by pathogens, reflecting excess radiation, and reducing water loss (Levin 1973; Symonds et al. 2005). Their functional role varies with plant species and growth environment. However, the function of leaf hairs in rice remains unknown.

Various genes related to leaf morphological traits in rice, including narrow leaf (*nal*), rolled leaf (*rl*), drooping leaf (*dl*), dripping-wet leaf (*drp*), and glabrous leaf (*gl*), have been identified, and their modes of inheritance have been elucidated in mutant lines (Kinoshita 1995). DNA markers have facilitated the genetic mapping of quantitative trait loci (QTLs) and the map-based cloning of genes for morphological and physiological leaf traits (Yonemaru et al. 2010). The rice hairy-leaf genes, *Hla* and *Hlb* (Nagao et al. 1960; Nagao and Takahashi 1963), also affect the hairiness of husks. Recently, a dominant gene for pubescence growth and development, *GL6*, was fine-mapped on chromosome 6 (Zeng et al. 2013).

In general, plant water use efficiency (WUE) is calculated as the amount of dry matter produced divided by the amount of water consumed by the plant during its growth (Condon et al. 2004), and photosynthetic WUE (WUE_p_) is defined as the amount of carbon fixed in photosynthesis per unit of water transpired (Hamid et al. 1990; Lawson and Blatt 2014). Plant WUE and WUE_p_ have a close relationship in some crop species (Heitholt 1989; Peng and Krieg 1992). Leaf morphological traits commonly affect physiological traits such as CO_2_ gas exchange and transpiration (Wright et al. 2004). Leaf hairs might influence photosynthesis (Johnson 1975; Pfeiffer et al. 2003). Although the physiological effects of leaf hairs in rice have been reported (Wada 2002), it is not well known how leaf hairs relate to photosynthetic traits or water use at the leaf level.

Here, we developed a chromosomal segment introgression line (IL) of the wild rice species *Oryza nivara* in the genetic background of *Oryza sativa* ‘IR24’ with a high leaf-hair density. Using genetic, physiological, and morphological analysis, we examined the effects of the leaf hairs on photosynthesis and WUE at the leaf level.

## Results

### A gene on chromosome 6 controls hairy-leaf trait of *O. nivara*

**Fig. 1:**
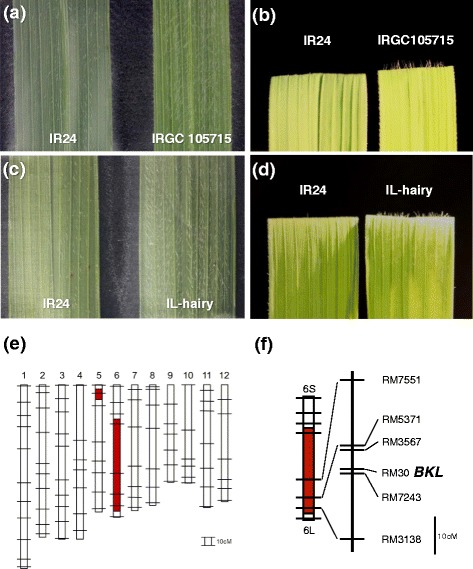
Phenotypes of leaf surface, graphical genotype of IL-hairy, and linkage map of *BKL* on chromosome 6. **a–d** Phenotypes of leaf hairs in vertical (**a, c**) and horizontal (**b, d**) view of IR24 and IRGC105715 (**a, b**) and IR24 and IL-hairy (**c, d**). **e** Graphical genotype of IL-hairy used for linkage analysis in BC_5_F_2_. Chromosomal segments derived from IRGC105715 and IR24 are indicated in *red* and *white*, respectively. **f** *Vertical bars* represent the positions of SSR markers. Numbers indicate map distances (cM) between SSR markers

*Oryza nivara* accession IRGC105715 had a high leaf-hair density (hairy leaf), whereas IR24 had few hairs (Fig. 1a, b). In the BC_4_F_4_ generation derived from a cross between IR24 and IRGC105715, one of the ILs showed the hairy-leaf trait from the sixth leaf to the flag leaf, and was named IL-hairy (Fig. 1c, d). To identify the chromosomal segments derived from IRGC105715, we determined the genotypes of the BC_4_F_3_ parent of IL-hairy using simple sequence repeat (SSR) markers covering the whole rice genome (Additional file 1: Table S1). IL-hairy had two segments derived from IRGC105715: at SSR marker *RM7302* on the short arm of chromosome 5 and from *RM8226* to *RM3138* on chromosome 6, all homozygous for the IRGC105715 regions (Fig. 1e). To identify the gene controlling leaf hairiness in IL-hairy, we conducted linkage analysis using 189 BC_5_F_2_ plants derived from backcrossing between IL-hairy and IR24. In this population, plants with hairy leaves and plants with normal (sparsely haired) leaves segregated in a 137:52 ratio. The 189 BC_5_F_3_ families derived from the BC_5_F_2_ individuals were classified into three groups: 36 populations in which all BC_5_F_3_ plants had the hairy-leaf phenotype, 101 populations in which hairy-leaf and normal-leaf plants segregated, and 52 populations in which all BC_5_F_3_ plants had the normal-leaf phenotype (Additional file 2: Table S2). The segregation fitted a 1:2:1 ratio, indicating that hairy leaf was controlled by a single dominant gene (χ^2^_1:2:1_ = 3.60, *P* = 0.17). We designated this gene *BLANKET LEAF* (*BKL*). Our linkage analysis using SSR makers on chromosome 6 revealed that *BKL* tightly linked to *RM30* (Fig. 1f). These data indicate that a single dominant gene tightly linked to *RM30* controls leaf hairiness in the BC_5_F_2_ and BC_5_F_3_ populations. *BKL* also located between SSR markers *RM3567* and *RM7243* at map distances of 5.8 and 1.1 cM, respectively (Fig. 1f).

### Morphological characteristics of leaf hair

**Fig. 2:**
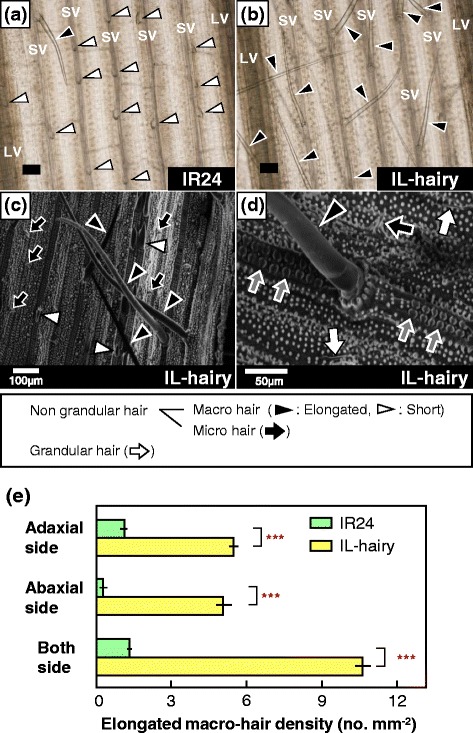
Light microscopic and scanning electronic microscopic images of leaf surface, and leaf hair density. **a, b** Light microscopic images of IR24 (**a**) and IL-hairy (**b**). *Scale bars*, 100 μm. **c, d** Scanning electronic microscopic images of adaxial surface of IL-hairy at tillering stage. *Black arrowheads*, elongated macro-hairs; *white arrowheads*, short macro-hairs; *black arrows*, micro-hairs; *white arrows*, glandular hairs; gray arrows, silica cells. LV, large vascular bundle; SV, small vascular bundle. **e** Density of elongated macro-hairs on adaxial side, abaxial side, and both sides in leaves of IR24 and IL-hairy at booting stage. Data are means ± SD (*n* = 3 or 4). ***P = 0.001 by Student’s *t*-test

In leaf blades of rice, macro-hairs array on silica cells over small vascular bundles, and micro-hairs and glandular hairs occur along stomatal rows or beside motor cells (Li et al. 2010, Fig. 2a-d). Leaves of IR24 had few elongated macro-hairs and many short macro-hairs along the small vascular bundles (Fig. 2a). In contrast, IL-hairy leaves had few short macro-hairs and many elongated macro-hairs along the small vascular bundles (Fig. 2b). The two types of macro-hair, short and elongated types, occurred over small vascular bundle (Fig. 2b–d).

At the booting stage, the densities of elongated macro-hairs on each leaf surface were much greater in IL-hairy than in IR24; the total density on both leaf surfaces in IL-hairy was 7.9 times that in IR24 (Fig. 2e). The lengths of elongated macro-hairs ranged between 300 and 400 μm in IR24 and between 500 and 800 μm in IL-hairy. In IR24 and IL-hairy, there was no difference in length between adaxial and abaxial surfaces (data not shown). Furthermore, at the tillering stage, total macro-hair (sum of elongated and short types of macro-hair) densities on both leaf surfaces did not different between two lines, although elongated macro-hair densities on both surfaces were also much greater in IL-hairy than in IR24 (Table 1). On the basis of the localization of macro-hairs and total macro-hair density, we considered that the elongated macro-hairs in IL-hairy are originated from short macro-hairs. In addition, hull hairiness showed no difference between IR24 and IL-hairy (Additional file 3: Fig. S1).

**Table 1:** Elongated macro-hair density and total macro-hair (sum of elongated and short types of macro-hair) density of leaves in IR24 and IL-hairy at the tillering stage

Line	Elongated macro-hair density (no mm^−2^)		Total macro-hair density (no mm^−2^)	
	Adaxial	Abaxial	Adaxial	Abaxial
IR24	0.1 ± 0.3	0.0 ± 0.0	15.2 ± 2.0	6.9 ± 2.3
IL-hairy	3.5 ± 0.5	3.8 ± 0.4	16.4 ± 1.5	7.0 ± 1.5
	*** ^a^	***	ns	ns

Data are means ± SD (*n* = 4). ^a^ *** *P* = 0.01; ns, not significant by Student’s *t*-test at *P* = 0.05

### Comparison of physiological and morphological traits between IL-hairy and IR24

**Fig. 3:**
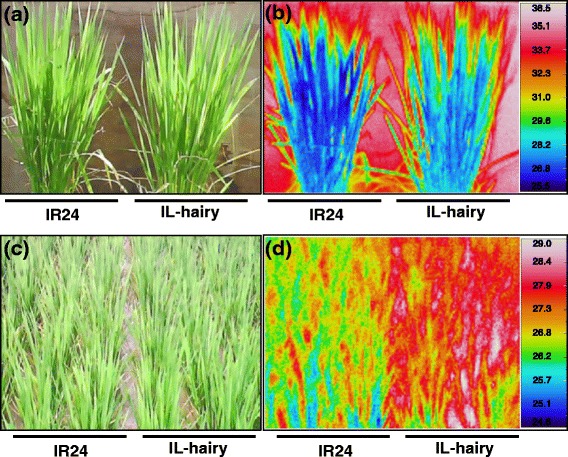
Visible and thermal images of IR24 and IL-hairy grown in pots (**a**, **b**) and in the field (**c**, **d**) at booting stage

**Fig. 4:**
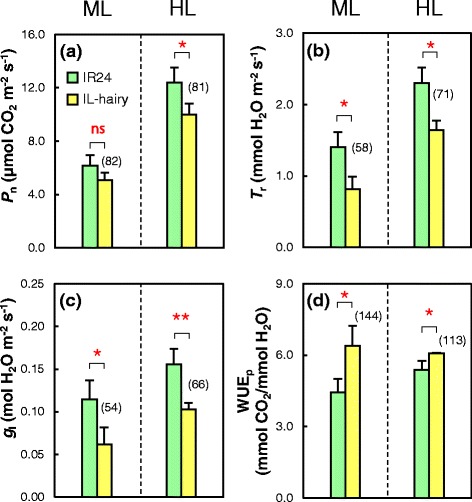
Photosynthetic traits in IR24 and IL-hairy. Net photosynthetic rate (*P*_n_; **a**), transpiration rate (*T*_r_; **b**), leaf diffusive conductance (*g*_l_; **c**), and photosynthetic water use efficiency (WUE_p_; **d**) under moderate (ML: 400 μmol m^−2^ s^−1^) and high light intensity (HL: 1500 μmol m^−2^ s^−1^) at booting stage. Data are means ± SD (*n* = 3 or 4). *P = 0.05, **P = 0.01; ns, not significantly different (*t*-test). Values in *parentheses* show percentage of IL-hairy values relative to IR24 values

Because the dense leaf hairs of IL-hairy would act as a blanket and thus affect leaf physiology, we measured daytime leaf surface temperatures. The leaf surface of IL-hairy was 1 to 2 °C warmer than that of IR24 at both plant (Fig. 3a, b) and canopy levels (Fig. 3c, d). We measured photosynthetic traits under a moderate light intensity (ML) of 400 μmol photons m^−2^ s^−1^ and a high light intensity (HL) of 1500 μmol photons m^−2^ s^−1^. Under HL, the net photosynthetic rate (*P*_n_), transpiration rate (*T*_r_), and leaf diffusive conductance (*g*_l_) of IL-hairy were significantly lower and WUE_p_ was significantly higher (by 13%) than those of IR24 (Fig. 4a–d). Under ML also, *P*_n_ was marginally lower, *T*_r_ and *g*_l_ of IL-hairy were significantly lower, and WUE_p_ was significantly higher (by 44%) than those of IR24. These data suggest that IL-hairy has lower photosynthetic activity but higher WUE_p_ than IR24.

We evaluated morphological and physiological leaf traits related to photosynthesis. In both lines, stomatal density was approximately 35% higher on the abaxial surface than on the adaxial surface, but there was no difference in stomatal density or guard cell length on either surface between the lines (Table 2). Specific leaf weight (SLW), as an indicator of leaf thickness, was not significantly different between lines. Values of chlorophyll, which is involved in photosynthetic rate by regulating energy production, and of leaf nitrogen, as a component of ribulose 1,5-bisphosphate carboxylase/oxygenase, were similar between lines (Table 2).

**Table 2:** Stomatal density and guard cell length, specific leaf weight, chlorophyll content, and nitrogen content of leaves at the booting stage in IR24 and IL-hairy

Line	Stomatal density (no. mm^−2^)		Guard cell length (μm)		Specific leaf wt. (g m^−2^)	Chlorophyll content (g m^−2^)	Nitrogen content (g m^−2^)
	Adaxial	Abaxial	Adaxial	Abaxial			
IR24	472 ± 16	625 ± 14	21.1 ± 0.7	20.8 ± 0.4	43.9 ± 1.0	0.41 ± 0.02	1.38 ± 0.05
IL-hairy	469 ± 9	646 ± 22	21.1 ± 0.4	20.9 ± 0.8	44.7 ± 1.2	0.39 ± 0.03	1.29 ± 0.06
	ns^a^	ns	ns	ns	ns	ns	ns

Data are means ± SD (*n* = 3–4). ^a^ ns, not significant by Student’s *t*-test at *P* = 0.05

**Fig. 5:**
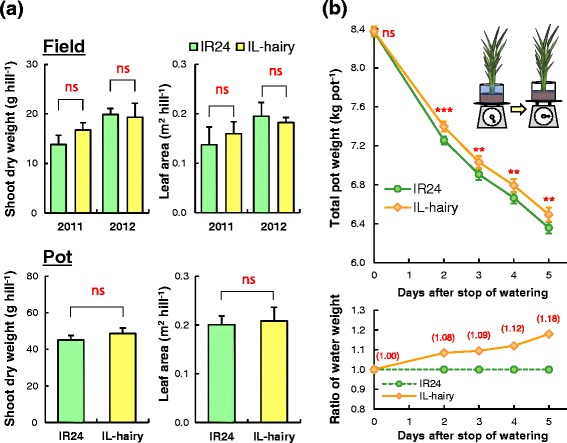
Shoot dry weigh, leaf area, and water uptake traits of the whole plant. **a** Shoot dry matter production and leaf area in the field (*vegetative stage*) and pots (*heading stage*). Data are means ± SD (field, *n* = 3; pot, *n* = 4). ns, not significantly different (*t*-test). **b** Changes of total *pot weight* including plant weight and ratio of water weight of IL-hairy relative to that of IR24 during 5 days after stop of watering at the booting stage. Data are means ± SD (*n* = 7). **P = 0.01, ***P = 0.001; ns, not significantly different (*t*-test). Values in parentheses show relative values of IL-hairy to IR24

Shoot dry weight and leaf area per hill did not differ significantly between lines at the vegetative stage in the field and at the heading stage in pots (Fig. 5a). In addition, other growth traits such as plant height, tiller number, leaf age, leaf SPAD value, and length and width of grain also did not significantly differ between lines (Additional file 4: Table S3, Additional file 3: Fig. S1). We also compared water diminution per a potted plant through transpiration during 5 days at the booting stage. As expected, IL-hairy showed significantly lower water diminution than IR24 from two to 5 days after stop of watering (Fig. 5b), and also ratio of water weight in the pot for IL-hairy to that of IR24 increased with the passage of time after stop of watering (Fig. 5b).

## Discussion

### Genetic analysis of hairy leaf gene *BKL*

Many studies of genotypic differences in plant growth and water use under drought, upland, and water-saving conditions have been carried out in rice. A large number of QTLs related to root traits under water-limited conditions have been identified (Zhang et al. 2001; Bernier et al. 2008; Kamoshita et al. 2008; Ding et al. 2011). QTLs for leaf water use and photosynthesis have been identified in rice by using carbon isotope discrimination values as an index (Price et al. 2002; Takai et al. 2009; Xu et al. 2009; This et al. 2010). However, there is little information on the genetic basis for high WUE. The hairy leaf trait offers a clue.

A number of QTLs and genes related to leaf hair development have been identified through the use of molecular markers in *Arabidopsis* (Larkin et al. 1999; Payne et al. 2000; Graham et al. 2006), wheat (Taketa et al. 2002; Dobrovolskaya et al. 2007), and soybean (Du et al. 2009). We identified *BKL* from IRGC105715 between markers *RM3567* and *RM7243* on chromosome 6, and it co-segregated with marker *RM30* (Fig. 1f). Among previously reported genes related to leaf hair development, we found *GL6*, related to pubescence growth and development, which was fine-mapped on chromosome 6 (Zeng et al. 2013), and *Hla*, related to long hair production, which was mapped on chromosome 6 by classical linkage analysis and may be complementary with *Hlb* (Nagao et al. 1960). The *BKL* locus might be the same as that of *GL6* or *Hla*.

### Predicted genes in *BKL* region

In the candidate region between markers *RM3567* and *RM7243*, 204 genes are annotated in the Nipponbare reference sequence in the Rice Annotation Project Database (RAP-DB, http://rapdb.dna.affrc.go.jp; Sakai et al. 2013) (Additional file 5: Table S4). Among them, five were possible candidates as the causal gene for *BKL*, since their homologs in *Arabidopsis thaliana* are associated with trichome formation.

*Os06g0646600* is classified in a class II KNOTTED-like homeobox (KNOX) subfamily of rice homeobox genes (Jain et al. 2008). The rice homeobox gene family is divided into zinc finger homeodomain (ZF-HD), homeodomain-leucine zipper protein (HD-ZIP), plant homeodomain (PHD) finger domain, Bell1-like homeobox (BLH), KNOX, and WUS-like homeobox (WOX) subfamilies (Jain et al. 2008). *Glabrous Rice 1*, which encodes a WOX, regulates micro-hair formation on rice leaves (Li et al. 2012). KNOX-type homeobox genes may be associated with the number of micro-hairs on rice leaves, although involvement of KNOX genes in trichome formation has not been reported in *Arabidopsis*.

*Os06g0649000* showed homology with a WRKY transcription factor (TF), *TRANSPARENT TESTA GLABRA2* (*TTG2*), in *Arabidopsis. TTG2* is strongly expressed in trichomes and acts downstream of the trichome initiation genes *TTG1* and *GLABROUS1*. A *TTG2*-disrupted mutant has no trichomes (Johnson et al. 2002).

*Os06g0649500* showed weak homology to *TTG1*, which encodes a WD40-repeat-containing protein. The TTG1 protein forms a complex with the R2R3-MYB TF GLABRA1 (GL1), the bHLH1 TF GLABRA3 (GL3), and a bHLH2 TF to promote trichome formation in *Arabidopsis*.

*Os06g0659100* and *Os06g0663500* were identified as *OsSPL10* and *OsSPL11* (*SQUAMOSA PROMOTER BINDING PROTEIN-LIKE* (*SPL*) genes in *O. sativa*), and phylogenetic analysis of *SPL* genes in rice and *Arabidopsis* placed *OsSPL10* and *AtSPL8* in group B, and *OsSPL11*, *AtSPL2*, *AtSPL10*, and *AtSPL11* in subgroup A2.2 (Yang et al. 2008). An *AtSPL8*-knockout mutant obtained by transposon tagging showed disrupted trichome formation (Unte et al. 2003). Transgenic plants expressing a dominant repressor version of *SPL10/11/2* formed many trichomes on their cauline leaves and flowers (Shikata et al. 2009).

Although other genes were also possible candidates, the above five genes were the most likely candidates on the basis of the information available at present.

### Morphological traits of leaf hair in IL-hairy

Leaf-hair density was obviously greater in IL-hairy than in IR24 (Figs. 1a–d, 2a, b, e). As the macro-hairs were located on silica cells over small vascular bundles (Fig. 2b–d) and total macro-hair densities on both leaf surfaces did not differ between lines (Table 1), we consider that elongated macro-hairs of IL-hairy are originated from short macro-hairs on small vascular bundles found in IR24, and *BKL* functions in the elongation of macro-hairs through epidermal cell differentiation. As dense leaf hairs were observed from the sixth-leaf stage, *BKL* acts at least from this stage.

On the other hand, glabrous rice varieties which show the reduction or absence of macro-hair on the surface of the hulls and leaves have been widely cultivated in several countries such as the United State and Australia (Rutger and Mackill 2001). These glabrous varieties are known to generate less dust during processing than pubescent varieties (Jodon 1965). In our observation, hull hairiness did not differ between IR24 and IL-hairy (Additional file 3: Fig. S1). Therefore, *BKL* does not affect macro-hair development on surface of hull.

Morphological traits of leaf hairs depend on species. For instance, *Arabidopsis* has branched hairs (trichomes) (Larkin et al. 1999), whereas wheat (*Triticum aestivum* L.) has long and short simple hairs (Taketa et al. 2002). It is well known that phytohormones control leaf hair development. In wheat, cytokinin and jasmonate (JA) increase the density of leaf hairs, and cytokinin also increases hair length (Kobayashi et al. 2012). In rhodes grass (*Chloris gayana* Kunth), JA increases the density of macro-hairs and affects trichome initiation (Kobayashi et al. 2010). JA ZIM-domain (JAZ) proteins repress JA-regulated anthocyanin accumulation and trichome initiation with interaction to the WD40-repeat/bHLH/MYB transcriptional complexes (Qi et al. 2011). These protein complexes contain GL1, GL3, TTG3, and EGL3 protein subunits, which showed homologous relationships to the predicted protein encoded in the candidate *BKL* region. As one or more phytohormones may participate in the promotion of leaf hair development by *BKL*, further studies will need to investigate them.

### Photosynthetic and growth traits of IL-hairy

IL-hairy provides a unique opportunity to examine the effects of a morphological character on physiological traits, because its genetic background is almost the same as that of IR24. IL-hairy had lower *P*_n_, *T*_r_, and *g*_l_ than IR24 under both moderate and high light intensities (Fig. 4a–c), but *T*_r_ and *g*_l_ were reduced more greatly than *P*_n_ in IL-hairy. As a result, WUE_p_ was higher in IL-hairy than in IR24 (Fig. 4d). We detected no differences in size and density of stomata, SLW, chlorophyll content, and nitrogen content of leaves between the two lines (Table 2). These facts indicate that *BKL* did not affect these morphological and physiological traits. Altogether, it is suggested that leaf hairs cause the increase in WUE_p_ through restraint of transpiration by increasing the boundary layer resistance. Shading sunlight by the hairs might partially reduce photosynthesis of IL-hairy. Generally, plants prevent excessive rise of leaf temperature by evaporative heat loss in the process of transpiration. Our results showed that leaf temperature at both plant and canopy levels was higher in IL-hairy than in IR24 (Fig. 3b, d). These results support the explanation that evaporative heat loss is reduced by the increased boundary layer resistance in IL-hairy. However, strict evaluation of boundary layer resistance in IL-hairy will be required. In addition, further studies should be made to know whether the relationship between WUEp and leaf hairiness is affected by environmental factors influencing Tr.

Shoot dry weight, leaf area, and other growth traits in both field and pot trials did not significantly differ between IL-hairy and IR24 (Fig. 5a, Additional file 4: Table S3). In the experiment on the change of pot weight after stop of watering, IL-hairy took up less water than IR24 (Fig. 5b). These results indicate that the leaf-hair trait mediated by *BKL* did not significantly affect biomass productivity, although *P*_n_ of IL-hairy was marginally lower than that of IR24 (Fig. 4a), and leaf hairs might improve not only WUE_p_ but also plant WUE.

We were able to determine the relationship between a morphological trait mediated by a single gene and physiological traits. The photosynthetic traits of IL-hairy, with a high WUE_p_, may be useful for improving drought tolerance and for reducing water inputs in rice cropping. The next step is to evaluate the effect of leaf hairiness on WUE_p_, plant WUE, and biomass productivity at the individual plant and canopy levels under water deficiency.

## Conclusions

We identified the *BLANKET LEAF* hairy-leaf gene from wild *Oryza nivara. BKL* is tightly linked to SSR marker *RM30* on chromosome 6, and appears to cause the elongation of macro-hairs. The hairy-leaf trait increased leaf surface temperature and WUE_p_ by restricting leaf transpiration. These traits may be useful in the development of rice cultivars adapted to water-saving cultivation systems.

## Methods

### Plant materials

A BC_1_F_1_ population was developed from a cross between *O. nivara* IRGC105715, an annual wild accession from Cambodia, and *O. sativa* ssp. *indica* ‘IR24’, as the recurrent parent (Additional file 6: Fig. S2). Plants were backcrossed to IR24 to develop the BC_4_F_1_ generation. Self-pollination was used to develop the BC_4_F_4_ generation. A BC_4_F_4_ introgression line (IL) with hairy leaves was named IL-hairy (Fig. 1c, d). IL-hairy was backcrossed with IR24 to develop the BC_5_F_1_ generation. The BC_5_F_1_ plants were self-pollinated, and 189 BC_5_F_2_ plants were used to detect the location of the gene for hairy leaf. The subsequent 189 BC_5_F_3_ lines were evaluated for leaf hairiness in a progeny test.

The BC_5_F_2_ and BC_5_F_3_ populations were grown in the paddy field of Kyushu University’s farm (33°37′N, 130°27′E) in the summer of 2013 and 2014, respectively. Each row contained 12 hills. Both populations were evaluated for leaf hairiness at the tillering stage. To evaluate physiological and morphological traits, we also grew plants outdoors in 8-L pots (one seedling per pot) filled with sandy loam in summer 2013. Pots received 1.0 g N as ammonium sulfate, 1.6 g P as calcium superphosphate, and 1.6 g K as potassium chloride as basal fertilizer; no additional fertilizer was applied. Sufficient water was supplied throughout.

### Genetic analysis

For genotyping using SSR markers, total genomic DNA was extracted from freeze-dried leaf tissue of the F_2_ population according to Dellaporta et al. (1983), with minor modifications. Polymerase chain reaction (PCR) analysis was performed in a 15 μL reaction mixture containing 50 mM KCl, 10 mM Tris · HCl (pH 9.0), 1.5 mM MgCl_2_, 200 μM each dNTP, 0.2 μM each primer, 0.75 units *Taq* polymerase (Takara, Otsu, Japan), and approximately 25 ng of template DNA in a GeneAmp PCR system 9700 (Applied Biosystems, Foster City, CA, USA). The thermal cycler was programmed for a first denaturation step of 5 min at 95 °C, followed by 35 cycles of 95 °C for 30 s, 55 °C for 30 s, and 72 °C for 30 s. PCR products were separated in 4% agarose gels (Agarose HT; Amresco Inc., Solon, OH, USA) in 0.5× TBE buffer. Chromosomal segments from IRGC105715 introgressed in the backcrossed progeny were detected using 93 rice SSR markers published in McCouch et al. (2002) and Chen et al. (1997) and one SSR marker originally designed in this study (Additional file 1: Table S1). Recombination values between markers were estimated by the maximum-likelihood equation (Allard 1956) using Kosambi’s mapping function (Kosambi 1944).

### Phenotyping of hairy-leaf trait in the field

At the tillering stage, individual BC_5_F_2_ plants grown in the field were directly examined to determine the presence of leaf hairs on the adaxial surface of the topmost fully expanded leaf on the main stem. Plants were scored as ‘normal-leaf’ if they had only a few hairs (IR24 type) or ‘hairy-leaf’ (IL-hairy type) for linkage analysis. The phenotypes of BC_5_F_3_ lines (IR24 type, segregating type, and IL-hairy type) were used for mapping of the hairy-leaf gene locus.

### Microscopic observations of leaf hairs and stomatal traits

Samples from the middle of leaf blades were fixed in formalin-acetic acid-alcohol (FAA) solution for at least 3 days. They were incubated in four changes of 70% ethanol at 80 °C for 12 h each, then in two changes of 80% lactic acid at 80 °C for 12 h, and finally cleared in saturated chloral hydrate solution for at least 24 h. The leaf hair and stomata on each side of the leaf were observed under a light microscope (Axioplan; Zeiss, Jena, Germany). Four square areas (1.5 × 4 mm^2^) on each surface were analyzed for the quantification of leaf hair density (50×), macro-hair density (50×), guard cell length (200×), and stomatal density (200×). Stomata were photographed with a digital camera (DXM1200F; Nikon, Tokyo, Japan), and 400× images of each surface in triplicate were used to measure guard cell length.

### Scanning electron microscopy

For scanning electron microscopic observation of leaf hairs, fully expanded leaves on the main stem grown in the field were sampled at 38 days after transplanting (tillering stage), washed in distilled water, air-dried, and sputter-coated with gold. The leaf surface was observed by scanning electron microscopy (JEOL JSM-5200; Mamiya Co., Ltd., Japan).

### Measurement of leaf temperature

Leaf temperature was measured with a portable infrared thermal camera (F30; NEC Abio, Tokyo, Japan) with a resolution of 0.1 °C and viewing angles of 28° horizontal and 21° vertical. In the field, the camera was set about 1 m above the sunlit leaf surface.

### Measurement of photosynthetic gas exchange and leaf sampling

Photosynthetic gas exchange of the topmost fully expanded leaf on the main stem of potted plants was measured with an open gas exchange system at the booting stage. The gas exchange rate was measured with a sandwich-type assimilation chamber, equipped with a fan for mixing air (PLC-4B; ADC, Hoddesdon, UK). The air temperature in the assimilation chamber was controlled by circulating temperature-controlled water to a radiator attached to the chamber. The air had been moisture-saturated at 21.5 C^o^ by a dew point generator before it was sent to the assimilation chamber. The air with controlled humidity was pumped into the chamber at a rate of 16.7 cm^3^ s^−1^, adjusted with a mass air flow regulator. The measurement was carried out under the conditions of 27.9 ± 0.4 °C in air temperature, 387 ± 3 μL CO_2_ L^−1^ in ambient CO_2_ concentration, and 68 ± 1.5% relative humidity in the chamber. The leaf area was 5.9 cm^2^. Light was provided by a metal halide lamp (LS-M180; Sumita Optical Glass Inc., Japan) at a photosynthetic photon flux density of 400 (ML) or 1500 μmol m^−2^ s^−1^ (HL). The CO_2_ concentration and water vapor pressure in the reference and sample air volumes were monitored with an infrared gas analyzer (Li-6262; LI-COR, Lincoln, NE, USA) and used to calculate *P*_n_ and *T*_r_ as described by Long and Hallgren (1985). WUE_p_ was calculated as *P*_n_ / *T*_r_. During measurement of gas exchange, leaf and air temperature were also measured by thermocouple for evaluating *g*_l_. *g*_l_ to water vapor transfer was calculated as described by Gaastra (1959).

Specific leaf weight, chlorophyll content, and nitrogen content of the same leaves used for gas exchange measurement were measured. The chlorophyll content of leaves was determined by a spectrophotometer (UV-1200; Shimadzu, Kyoto, Japan) according to the method of Wintermans and de Mots (1965). Leaf samples were oven-dried at 80 °C for at least 2 days and weighed. The leaf nitrogen content of dried samples was quantified by a mass spectrometer (ANCA-SL; Europa Scientific, Franklin, USA).

### Growth traits and water uptake

To evaluate dry matter production and leaf area, IL-hairy and IR24 were grown in the paddy field (one seedling per hill; spacing 25 cm × 25 cm) in 2011 and 2012. Seedling at fifth-leaf stage were transplanted at a rate of 16 hills m^−2^ (spacing 25 cm × 25 cm) with one plant per hill. As a basal fertilizer, chemical fertilizer was applied at a rate of 5.0 g N, 12.0 g P and 12.0 g K m^−2^ in form of ammonium sulfate, calcium superphosphate and potassium chloride, respectively. The plots were randomly arranged with three replicates. Plants were also grown outdoor in 8-L pots filled with paddy soil (5.0 kg DW) and compound fertilizer containing 0.35 g N, 0.35 g P and 0.35 g K. Plant shoots were sampled at 28 days after transplanting in the field experiment and at heading stage in the pot experiment. Subsequently, the leaf area was measured by use of an automatic area meter (AAM-8; Hayashi-denko, Tokyo, Japan), and then the samples were oven-dried at 80 °C for 3 days to determine dry weight. In addition, plant height, tiller number, leaf age, and SPAD value (SPAD-502; Konica Minolta Sensing, Japan) were measured at the 28 days after transplanting in 2011. For evaluating grain phenotypes, grains of IR24 and IL-hairy were photographed with digital camera and measured grain length and width by caliper.

To compare water diminution by transpiration from plant between IL-hairy and IR24, plants were grown in 8-L pots filled with paddy soil (5.0 kg DW) containing 1.0 g N, 0.5 g P and 0.5 g K in form of urea, calcium superphosphate and potassium chloride, respectively, in a greenhouse under natural sunlight. Water diminution per pot was evaluated at booting stage. Total pot weight including plant was adjusted 8.4 kg by water (approx. 2.8 kg). Then, plant weight was approx. 0.3 kg. Subsequently, the plants of pots were withheld for irrigation during five consecutive clear days. Pot weight was measured every day at the same time to estimate the water diminution (the total amount of transpiration from the plant and evaporation from water surface of the pot) per day. In this estimation, we postulated that the water loss from water surface is the same in all pots of IL-hairy and IR24. As there were no significant differences in shoot dry weight and leaf area between the two lines (Fig. 5a), the difference in total pot weight between the two lines reflects that in the leaf-hair development. Water weight per pot was estimated by subtracting weight of soil, pot, and plant from total pot weight, and ratio of water weight of IL-hairy relative to that of IR24 was calculated.

## Additional files

Additional file 1: **Table S1.** List of primers for SSR markers used in this study. (XLSX 25 kb)

Additional file 2: **Table S2.** Segregation of the hairy leaf trait in the BC_5_F_3_ populations derived from a cross between IR24 and IL-hairy. (XLSX 16 kb)

Additional file 3: **Figure S1.** Grain phenotypes. **a** Phenotype of hull surface in IR24 and IL-hairy. Scale bar, 5 mm. **b**, **c** Grain length (**b**) and grain width (**c**) of IR24 and IL-hairy. Data are means ± SD (*n* = 10). ns, not significantly different (*t*-test). (PPTX 107 kb)

Additional file 4: **Table S3.** Growth traits of IR24 and IL-hairy grown under the field at the tilling stage. (XLSX 16 kb)

Additional file 5: **Table S4.** Gene annotation by RAP-DB in BKL region. (XLSX 29 kb)

Additional file 6: **Figure S2.** Breeding scheme for the plant materials used in this study. (PPTX 38 kb)

## Abbreviations

BLH: Bell1-like homeobox
DW: Dry weight
g_l_: Leaf diffusive conductance
HD-ZIP: Homeodomain-leucine zipper protein
HL: High light intensity
IL: Introgression line
JA: Jasmonate
KNOX: KNOTTED-like homeobox
ML: Moderate light intensity
PHD: Plant homeodomain
P_n_: Net photosynthetic rate
QTL: Quantitative trait locus
SLW: Specific leaf weight
SSR: Simple sequence repeats
T_r_: Transpiration rate
WOX: WUS-like homeobox
WUE: Water use efficiency
WUEp: Photosynthetic water use efficiency
ZF-HD: Zinc finger homeodomain
