# Supplementary material for: A hairy-leaf gene, BLANKET LEAF, of wild Oryza nivara increases photosynthetic water use efficiency in rice
Source: Rice (N Y). 2017 May 12;10:20. doi: 10.1186/s12284-017-0158-1 (PMC5429320; doi:10.1186/s12284-017-0158-1)
Supplement: Supplementary file 3 — Figure S1. Grain phenotypes. a Phenotype of hull surface in IR24 and IL-hairy. Scale bar, 5 mm. b, c Grain length (b) and grain width (c) of IR24 and IL-hairy. Data are means ± SD (n = 10). ns, not significantly different (t-test). (PPTX 107 kb) [file 12284_2017_158_MOESM3_ESM.pptx]

## Slide 1
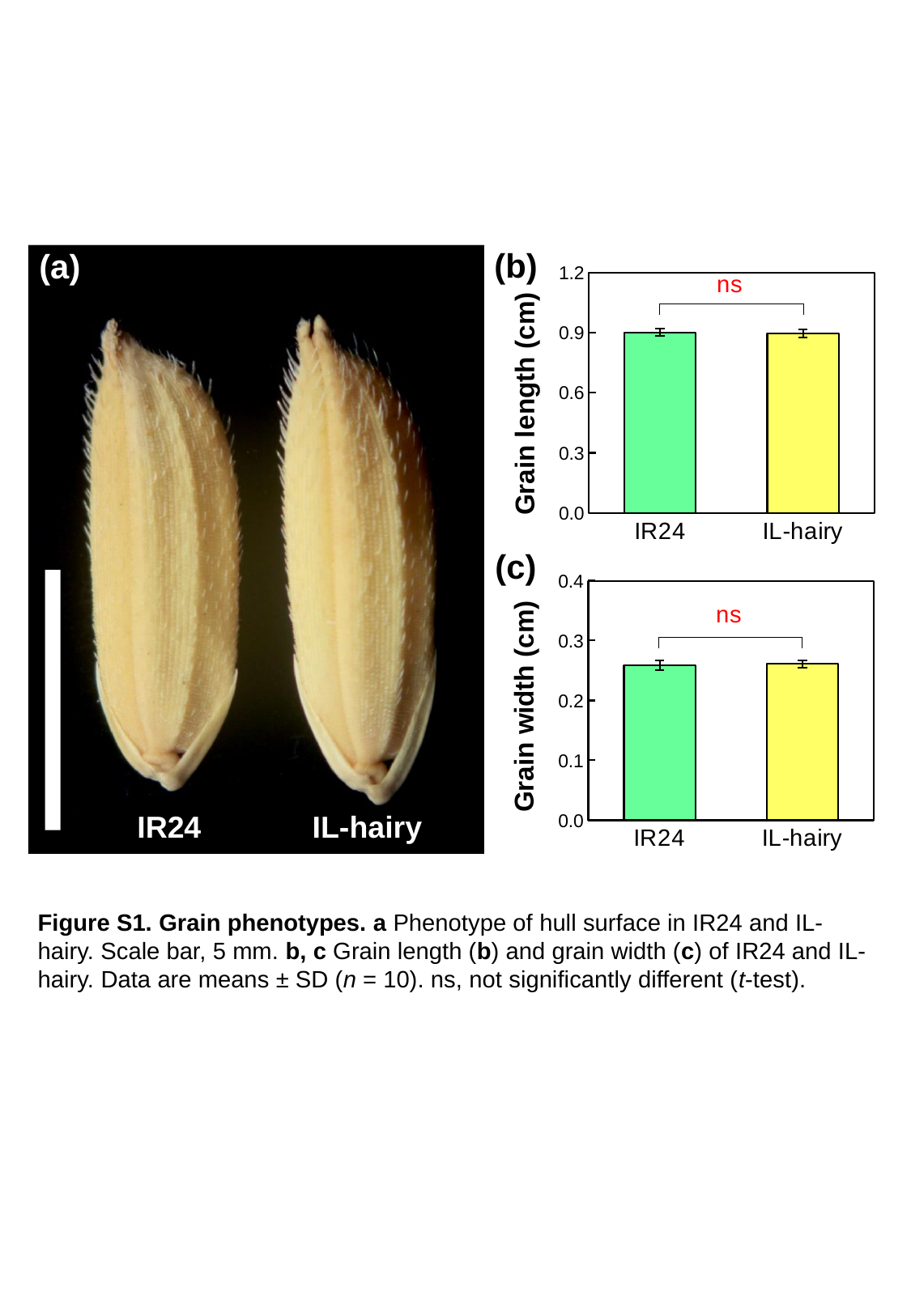

### Chart
| Category | |
|---|---|
| IR24 | 0.9015000000000001 |
| IL-hairy | 0.8955 |(b)
(a)
IR24
IL-hairy
(c)
### Chart
| Category | |
|---|---|
| IR24 | 0.25850000000000006 |
| IL-hairy | 0.261 |Figure S1. Grain phenotypes. a Phenotype of hull surface in IR24 and IL-hairy. Scale bar, 5 mm. b, c Grain length (b) and grain width (c) of IR24 and IL-hairy. Data are means ± SD (n = 10). ns, not significantly different (t-test).
