# Supplementary material for: A hairy-leaf gene, BLANKET LEAF, of wild Oryza nivara increases photosynthetic water use efficiency in rice
Source: Rice (N Y). 2017 May 12;10:20. doi: 10.1186/s12284-017-0158-1 (PMC5429320; doi:10.1186/s12284-017-0158-1)
Supplement: Supplementary file 6 — Figure S2. Breeding scheme for the plant materials used in this study. (PPTX 38 kb) [file 12284_2017_158_MOESM6_ESM.pptx]

## Slide 1
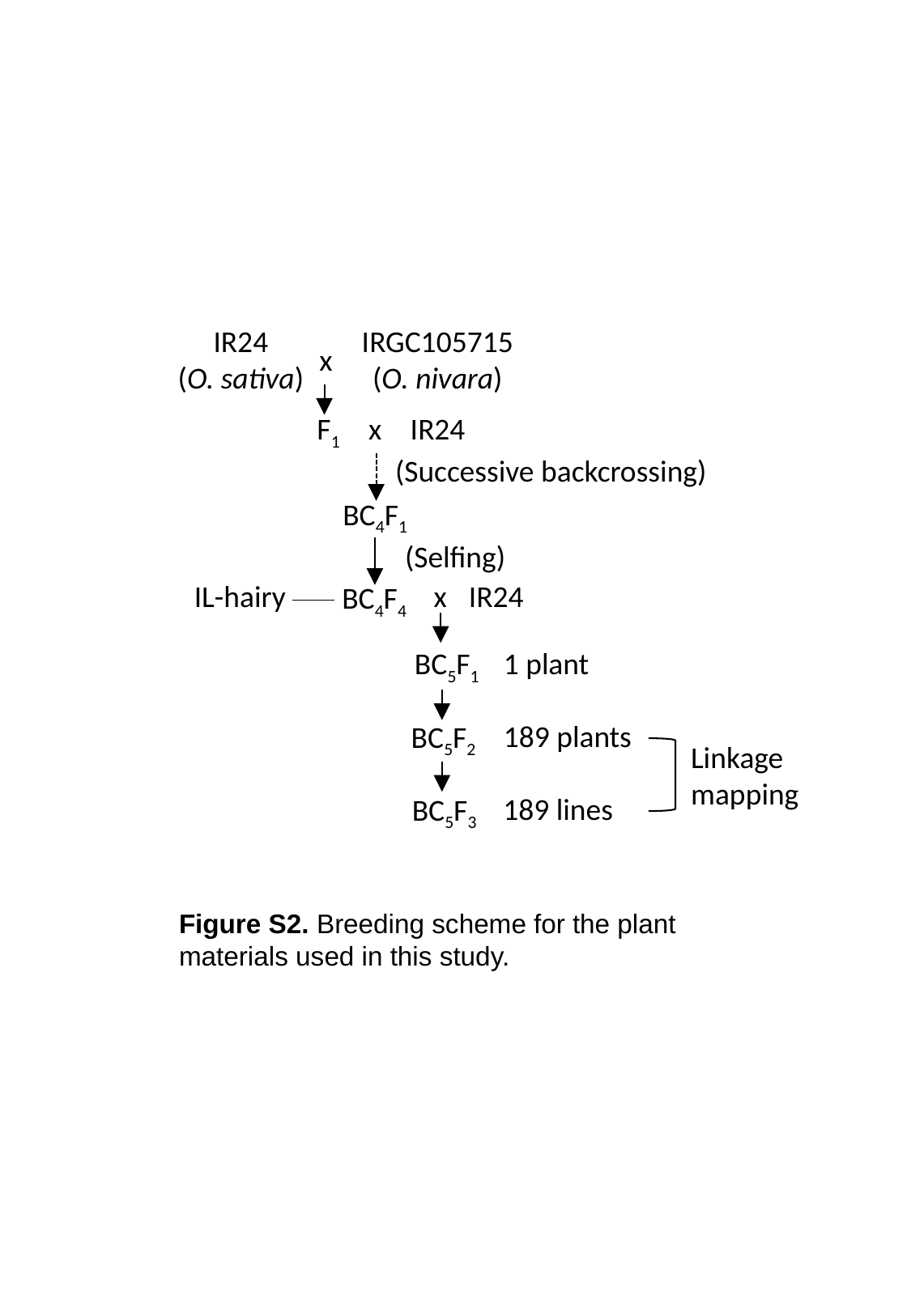

IR24
(O. sativa)
IRGC105715
(O. nivara)
x
F1
x
IR24
(Successive backcrossing)
BC4F1
(Selfing)
IL-hairy
x
IR24
BC4F4
1 plant
BC5F1
189 plants
BC5F2
Linkage
mapping
189 lines
BC5F3
Figure S2. Breeding scheme for the plant materials used in this study.
